# An Interdisciplinary Collaboration to Improve Hypertension Medication Adherence for Older Adults

**DOI:** 10.1093/geroni/igab046.879

**Published:** 2021-12-17

**Authors:** Jeannie Lee, Wendy Rogers

## Abstract

Hypertension is highly prevalent in older adults (74.5% in ≥60 years) with dire consequences, and adherence to hypertension medications is low (approximately 50%). With increased smartphone use among older adults (81% for 60-69 years, 62% for ≥70 years), technology innovations can improve medication adherence. This symposium highlights the efforts of an innovative interdisciplinary team of experts (clinical, cognitive aging, human factors, health technology) to develop and implement the Medication Education, Decision Support, Reminding, and Monitoring (MEDSReM) system to improve hypertension medication adherence for older adults. MEDSReM is a theory-based, integrated mobile application (app) and companion web portal that educates, supports missed dose decisions, reminds, monitors adherence, and incorporates blood pressure feedback. In this symposium, we describe the interdisciplinary development efforts. Insel et al. will present the theory-based intervention, technology translation, and advancement of the MEDSReM system. Lee et al. will describe the interdisciplinary team and describe the work by the decision support subteam that created the medication formulary and generated an algorithm to guide missed-dose decisions based on pharmacology of aging. Rogers et al. will discuss the education subteam’s development of educational information about hypertension, medications, and adherence for the MEDSReM system. Mitzner et al. will illustrate the instructional support sub-team’s efforts to ensure older adults can interact with both the smartphone app and online portal. Lastly, Hale et al. will describe the user testing subteam’s usability processes including the integration of blood pressure self-monitoring. These efforts will provide insights for other interdisciplinary teams developing technology interventions for older adults.

